# Unblock the Block! Preventing Inhibitory Failure to Maintain Inhibitory Restraint

**DOI:** 10.1177/15357597221098808

**Published:** 2022-07-16

**Authors:** Gabriele Lignani

**Affiliations:** 1 UCL Queen Square Institute of Neurology, UK

## Abstract

**Disrupting Epileptiform Activity by Preventing Parvalbumin Interneuron Depolarization Block**

Călin A, Ilie AS, Akerman CJ. *J Neurosci*. 2021;41(45):9452-9465. doi:10.1523/JNEUROSCI.1002-20.2021. Epub 2021 Oct 5.

Inhibitory synaptic mechanisms oppose epileptic network activity in the brain. The breakdown in this inhibitory restraint and propagation of seizure activity has been linked to the overwhelming of feedforward inhibition, which is provided in large part by parvalbumin-expressing (PV) interneurons in the cortex. The underlying cellular processes therefore represent potential targets for understanding and preventing the propagation of seizure activity. Here we use an optogenetic strategy to test the hypothesis that depolarization block in PV interneurons is a significant factor during the loss of inhibitory restraint. Depolarization block results from the inactivation of voltage-gated sodium channels and leads to impaired action potential firing. We used focal NMDA stimulation to elicit reproducible epileptiform discharges in hippocampal organotypic brain slices from male and female mice and combined this with targeted recordings from defined neuronal populations. Simultaneous patch-clamp recordings from PV interneurons and pyramidal neurons revealed epileptiform activity that was associated with an overwhelming of inhibitory synaptic mechanisms and the emergence of a partial, and then complete, depolarization block in PV interneurons. To counteract this depolarization block, we developed protocols for eliciting pulsed membrane hyperpolarization via the inhibitory opsin, archaerhodopsin. This optical approach was effective in counteracting cumulative inactivation of voltage-gated channels, maintaining PV interneuron action potential firing properties during the inhibitory restraint period, and reducing the probability of initiating epileptiform activity. These experiments support the idea that depolarization block is a point of weakness in feedforward inhibitory synaptic mechanisms and represents a target for preventing the initiation and spread of seizure activity.

## Commentary

Epilepsy is characterised by seizures, and therefore a deep understanding of the mechanisms of seizure initiation and propagation is pivotal for the field. The role of inhibitory neurons in seizure initiation is among the most intensely studied.^1^ It is well known that inhibitory activity restrains overexcitation, and that failure of this protective mechanism is central to seizure initiation.^2–4^ There are several reasons, still debated, why inhibitory restraint can fail. One potential mechanism is that accumulation of chloride ions in neurons innervated by intensely firing interneurons shifts the reversal potential for GABA (E_GABA_) in a depolarizing direction, thereby attenuating the inhibitory action of GABA or even converting it to excitation. A further consequence of intracellular chloride accumulation is that it leads to an outward flux of potassium ions carried by the potassium-chloride co-transporter. Accumulation of extracellular potassium can then lead to depolarization of excitatory neurons. A further potential mechanism is that interneurons themselves are over-depolarized, because they receive an intense barrage of glutamatergic excitation, possibly exacerbated by the effect of extracellular potassium accumulation.^1^ Such over-depolarization prevents interneurons from firing repetitively because sodium channels eventually become inactivated, a phenomenon known as ‘depolarization block’, and as a consequence GABA release fails. When depolarization block occurs, further action potentials cannot be triggered and thus interneurons become silent, leading to an escape of excitatory neurons from the inhibitory restraint. This might result in the triggering of the seizure. Several studies have provided evidence, in different pre-clinical models, for each of these mechanisms, which are not mutually exclusive.^5,6^ However, a direct test of causality for any of these hypotheses is not trivial.

In their recent publication, Călin and colleagues used an elegant experimental design to determine whether depolarization block of parvalbumin-positive (PV) interneuron accompanies seizure initiation.^7^ They used an established in vitro model based on NMDA-evoked epileptiform discharges (EDs) in organotypic hippocampal cultures. This model, which preserves aspects of the anatomy of the hippocampus, allows repeated EDs to be elicited.^7^ Călin et al performed both voltage and current clamp recording in PV interneurons and pyramidal cells in the CA1 sub-field, while pressure-applying NMDA to CA3.

Firstly, they showed that, while NMDA is applied, a pre-ED period can be detected during which inhibitory constraint is still active, but that this fades away before the initiation of a full-blown ED.^4,6,7^ This is accompanied by a decrease in both the frequency and the amplitude of action potentials recorded from PV interneurons, consistent with impending depolarization block.^8^ To test whether the decrease in spike frequency and amplitude was indeed because of over-depolarization, Călin et al expressed the hyperpolarizing opsin archaerhodopsin (Arch). Trains of short light pulses designed to hyperpolarize the membrane potential, and thereby release sodium channels from inactivation,^7^ during the pre-ED period led to an increase in spike frequency and amplitude, consistent with rescue from depolarization block. Furthermore, this manipulation was sufficient to decrease the probability of initiation of EDs without interfering with their onset delay and morphology.^7^ This observation suggests that depolarization block of PV positive interneurons in the critical period before an ictal event is indeed a mechanism by which the inhibitory constraint can fail, thereby triggering seizure initiation.

This study is arguably the most direct test of causality available, to show how depolarization block in PV expressing interneurons can lead to seizure initiation. This had been postulated and tested in previous studies, but never directly.^1^ The importance of the advance reported by Călin and colleagues is that it highlights the potential to tailor future treatments to counteract depolarization block in interneurons with spatial and temporal control, for example by using closed-loop optogenetics or other advanced manipulations. Another potential therapeutic avenue may be to use gene or RNA therapy to overexpress sodium channel splice variants with faster recovery from inactivation.^9^ This latter approach would have to be targeted specifically to interneurons, and would need to be studied closely to determine whether they could interfere with physiological interneuron activity. For example, although a *SCN1A* splice variant with faster recovery from inactivation has been lost during evolution, probably to protect from a gain of function effect, in the case of a pathological hyperactivity condition such as epilepsy, its reintroduction in interneurons may be therapeutic.

Depolarization block of PV-positive interneurons may not be the only event occurring in the lead-up to seizure initiation, and it will be important to understand how it interacts with other mechanisms. A potential limitation of the study by Călin et al is that the data were obtained in a model of acutely evoked seizures in vitro. To be verified in vivo*,* a model of chronic epilepsy with spontaneous seizures is necessary, with all the technical difficulties of recording and manipulating membrane potentials in the intact brain. A systematic experimental design to test different hypotheses underlining the failure of inhibitory restraint in the same in vivo model could be a significant step forward in the field. Nevertheless, the work by Călin and colleagues opens new avenues for the understanding of epilepsy and seizure initiation, as well as for developing new potential therapeutic approaches to stop seizure initiation and spreading.
